# Decoding Heavy Metal Stress Signalling in Plants: Towards Improved Food Security and Safety

**DOI:** 10.3390/plants9121781

**Published:** 2020-12-16

**Authors:** Marshall Keyster, Lee-Ann Niekerk, Gerhard Basson, Mogamat Carelse, Olalekan Bakare, Ndiko Ludidi, Ashwil Klein, Lukhanyo Mekuto, Arun Gokul

**Affiliations:** 1 Environmental Biotechnology Laboratory, Department of Biotechnology, University of the Western Cape, Bellville 7535, South Africa; 3255882@myuwc.ac.za (L.-A.N.); 3341863@myuwc.ac.za (M.C.); 3779970@myuwc.ac.za (O.B.); 2 DST-NRF Centre of Excellence in Food Security, University of the Western Cape, Bellville 7530, South Africa; nludidi@uwc.ac.za; 3 Plant Biotechnology Research Group, Department of Biotechnology, University of the Western Cape, Bellville 7535, South Africa; gerhardbasson94@gmail.com; 4 Plant Omics Laboratory, Department of Biotechnology, University of the Western Cape, Bellville 7535, South Africa; aklein@uwc.ac.za; 5 Department of Chemical Engineering, University of Johannesburg, Johannesburg 2028, South Africa; lukhanyom@uj.ac.za

**Keywords:** cell wall signaling, food safety, food security, glutathione, heavy metals, hydrogen sulfide, reactive oxygen species signaling

## Abstract

The mining of heavy metals from the environment leads to an increase in soil pollution, leading to the uptake of heavy metals into plant tissue. The build-up of toxic metals in plant cells often leads to cellular damage and senescence. Therefore, it is of utmost importance to produce plants with improved tolerance to heavy metals for food security, as well as to limit heavy metal uptake for improved food safety purposes. To achieve this goal, our understanding of the signaling mechanisms which regulate toxic heavy metal uptake and tolerance in plants requires extensive improvement. In this review, we summarize recent literature and data on heavy metal toxicity (oral reference doses) and the impact of the metals on food safety and food security. Furthermore, we discuss some of the key events (reception, transduction, and response) in the heavy metal signaling cascades in the cell wall, plasma membrane, and cytoplasm. Our future perspectives provide an outlook of the exciting advances that will shape the plant heavy metal signaling field in the near future.

## 1. Introduction

“Heavy metals” as a term has been widely debated and criticized. However, Duffus [1] reviewed the concept, and proposed that the scientific world come up with a standard definition. Heavy metals occur naturally in the Earth’s crust [2] and are extensively mined in various parts of the world. This practice has hugely contributed to the uncontrollable anthropic inputs into the environment. These heavy metals have a very long half-life, and therefore persist in the environment for extended periods, because they cannot be degraded [3,4]. Therefore, they can accumulate and exert toxic effects on microorganisms, plants, animals, and humans. Heavy metals have become a major environmental issue, and if the current environmental contamination persists, then heavy metal pollution will also be a great concern for the future [5].

Heavy metals such as arsenic, cadmium, chromium, lead, and mercury rank among the most toxic metals, with high public health significance. Toxicity in humans and animals often depends on several factors, such as quantity, route of exposure, and chemical species. However, age, gender, genetics, and nutritional status also play key roles in physiological responses to heavy metals. Humans and animals can come into contact with heavy metals through rocks, soils, water, and the atmosphere [6]. Plants utilize all of the mentioned resources to survive, and due to a sessile nature cannot avoid contaminated environments. Therefore, these plants take up the heavy metals and sequester the metals in various tissues, maintaining heavy metal concentrations below toxicity levels [7]. Humans and animals which consume heavy metal contaminated plants can accumulate them to toxic levels in various organs [8]. Therefore, it is important to understand the pathways that control heavy metal uptake in plants. This will assist in regulating tolerance to, and monitoring toxicity of, heavy metals in order to limit the risk that results from consumption of plants contaminated with heavy metals.

Plants have evolved complex signaling mechanisms that regulate their responses to heavy metal stress [9]. These mechanisms include the “universal” cascade, which consists of reception (stimuli perception), transduction (intra and extracellular signal amplification), and response (enzymatic or non-enzymatic) steps [10]. Therefore, in this review we focus on some of the key events at each step of the plant signaling cascade under heavy metal stress. We discuss the importance of the apoplastic space in the reception of heavy metals, especially the roles of the cell wall and the plasma membrane (PM). We discuss the complexity of reactive oxygen species (ROS) signaling in plants under heavy metal stress. We review and discuss the key importance of glutathione and hydrogen sulfide, in heavy metal detoxification, as well as serving as possible heavy metal stress signal amplification molecules. In addition, we also examine the latest US Environmental Protection Agency [11] data about key heavy metals, in order to understand toxicity for food safety purposes by analyzing the oral reference doses (RfDo) for the heavy metals (Table 1).

## 2. Heavy Metals in Crop Plants Contributes to Food Safety Risks

The food safety risks associated with consumption or exposure to heavy metals have been widely reported and reviewed [14,15,16]. Mosby et al. [17] reported that there are approximately 35 toxic metals which are of concern to humans. Twenty (Table 1) out of the 35 metals are classified as “heavy” metals, as extrapolated from [11]. The term “heavy metal” is widely debated, therefore we included only metals with a density at room temperature of more than 5 gm/cm^3^. This group includes: antimony, arsenic, cadmium, chromium, cobalt, copper, iron, lead, manganese, mercury, molybdenum, nickel, silver, thallium, tin, tungsten, uranium, vanadium, zinc, and zirconium. Our analysis shows that the top five metals (Table 1), ranked according to elemental abundance, are iron (4th), manganese (12th), chromium (21st), zirconium (18th), and vanadium (19th). Furthermore, the top five heavy metals, ranked according to the minimum limit in residential soils, are chromium, arsenic, thallium, zirconium, and mercury (Table 1). Based on the two ranking systems, chromium and zirconium are both abundant but also very poisonous to humans. The ranking based on RfDo, which is the recommended minimum intake of the heavy metals per day (µg/kg human), is thallium, zirconium, uranium, arsenic, and cobalt. Data shows that zirconium is an abundant (in the environment) and very toxic heavy metal when consumed by humans. Very few studies include thallium and zirconium in environmental and soil surveys, which is extremely worrying. Wierzbickae et al. [18] reported high levels of thallium in a 100-year-old calamine waste heap in Olkusz (southern Poland). The authors reported that the highest value of thallium in the soils was 78 mg/kg. The study also included a screening of thallium in four plants which grew in the vicinity, and found that *Plantago lanceolata* accumulated extremely high amounts of thallium in the roots (maximum of 321 mg/kg) and shoots (maximum of 180 mg/kg). Animals such as sheep often feed on *P. lanceolata* [19], and therefore the thallium could accumulate in the animals and subsequently be transferred to humans. *P. lanceolata* is also included in herbal teas [20], and when prepared the thallium could be released from the tea into the human digestive system. Queirolo et al. [21] conducted experiments to test the level of thallium in potato, broad bean, and maize plants in the El Loa province of Chile. Inductively coupled plasma-mass spectrometry (ICP-MS) data showed that potatoes accumulated the highest amounts of thallium (1.8–4.1 mg/kg), which exceeds the RfDo value prescribed by the USEPA. This study highlights the concern with the current school of thought that leafy vegetables should be classified as high risk for heavy metal transfer to humans. Root vegetables are in direct contact with the heavy metals (in soils), and therefore should also be consumed with extreme care, especially when obtained from a region in close proximity to mines. Biata et al. [22] confirmed this notion when they conducted inductively coupled plasma-optical emission spectroscopy (ICP-OES) studies on different vegetables consumed in Johannesburg, South Africa. This study found more thallium in potatoes (1.04 mg/kg) than in spinach (0.318–0.322 mg/kg).

Very few studies have included zirconium in soil surveys, and even fewer have focused on zirconium levels in plants. This is a serious knowledge gap that requires urgent attention by the scientific community, since zirconium is listed as the second most toxic heavy metal element by the USEPA. Malandrino et al. [23] conducted a survey of heavy metals in northeast Piedmont (Italy), and included zirconium in the study. The authors found up to 103 mg/kg of zirconium in the soils, but did not include the metal in further pot experiments to screen plant responses. A few studies have shown that zirconium accumulates more in roots than in the shoots. Shi et al. [24] showed that Zirconium-95 (^95^Zr) accumulates more in the roots of *Oryza sativa* than in its shoots. This finding is very important because the ^95^Zr isotope, which is released into the environment at the highest amounts upon nuclear accidents, can be readily absorbed by plants [25]. Furthermore, Fodor et al. [26] conducted a pot experiment to test the uptake of zirconium in wheat. When the plants were treated with 550 μM of zirconium, they accumulated more zirconium in the roots (~1400 mg/kg) than in the shoots (~200 mg/kg). An important study was done by Shi and Guo [27] on *Brassica rapa* subsp. *pekinensis* treated with ^95^Zr. The authors conducted pot experiments which showed that zirconium moved throughout the entire plant in 6 h. The study showed that zirconium accumulated more in the roots than the leaves of the *B. rapa* subsp. *pekinensis*. The study by Shi and Guo [27] abolished the notion that zirconium is not transported to the aerial parts of plants.

Even though this section mainly focused on the top two most toxic heavy metals, thallium and zirconium, the toxicity and risk to human health of the other heavy metals (in the top five) should not be ignored. The toxicity of the other three metals (in the top five) was previously discussed by Baumann et al. [28] and Gupta et al. [29] (uranium), Ha et al. [30] (arsenic), and Hu et al. [31] and Kosiorek and Wyszkowski [32] (cobalt). According to the USEPA (Table 1), 0.8 µg of thallium per day is allowed for an 80 kg person. Therefore, if an 80 kg person prepares a meal containing 100 g of potato, which has accumulated approximately 100 µg of thallium [22], this meal would be 125 times over the legal limit of the USEPA, and this person would be seriously poisoned. Due to the higher RfDo values of the most studied heavy metals, the potential threat of heavy metal poisoning has often been downplayed as only being dangerous after exposure over long periods of time [33]. However, the potential risk of thallium and zirconium toxicity should escalate the importance of heavy metal toxicity from crop consumption.

## 3. Heavy Metal Pollution Decreases Food Security

Food security as a term has various meanings, with various definitions [34]. However, a global definition was agreed upon at the 2001 the Food and Agriculture Organization of the United Nations (FAO) World Food Summit: “Food security exists when all people, at all times, have physical, (social) and economic access to sufficient, safe and nutritious food which meets their dietary needs and food preferences for an active and healthy life” [35]. Simón [36] highlighted four dimensions to food security, namely food availability, food access, food utilization, and the stability of food availability, access, and utilization. Heavy metal stress, which directly affects food crops, has detrimental impacts on food access (amount of food) and food utilization (safety and nutrition). The impacts of heavy metal stress on food safety enjoy much of the attention, but very little is reported on the impacts of heavy metals on food access (crop yields from fields) and nutrition (impact on important dietary nutrients). Fu et al. [37] reported that the collapse of the tailing dam of the Beishan lead–zinc mine, which is located upstream of Huanjiang river (China), led to widespread contamination of the surrounding farmlands. Some of the surrounding regions could no longer sustain crop production because of very serious pollution, and the lesser affected regions could produce agricultural products, but with poor quality [38]. The region surrounding the river had high concentrations of arsenic and lead, which were all above the national permissible standards according to China’s Environmental Quality Standard for Soils (GB15618-1995, Grade II) for farmland soil, but cadmium concentrations were lower than this national standard [38]. In a previous report, Clemente et al. [39] showed that the soil at this experimental site was highly contaminated by zinc, copper, and lead, with a wide range of pH values. Clemente et al. [39] reported that a mine spill at Aznalcόllar (Seville, Spain) caused serious contamination along the Guadiamar river, as well as in surrounding arable lands. Further studies in the area by Grimalt et al. [40] showed that the soil contained high levels of zinc, copper, and lead, with a wide range of pH values. Clemente et al. [41] conducted a study using the area as an experimental station to evaluate the potential of *Brassica juncea* for phytoextraction of the heavy metals. The authors reported that *B. juncea* did not grow homogeneously in the experimental site, and that the first *B. juncea* crop, growing without fertilizer and manure treatments, produced very low biomass. Meers et al. [42] conducted field experiments in the historically contaminated region of Campine (along the Dutch–Belgian border) on six cultivars of maize. The contamination in the region is due to non-ferro smelter activity, and high levels of cadmium, lead, and zinc have been reported in surrounding soils. The area is mainly used for agriculture, even though crops produced in the region often exceed European food and feed standards, mainly for cadmium and zinc. The authors reported that the biomass (FW) of maize ranged between 36,000 and 52,000 kg/ha for the six cultivars, which was 60%–74% lower than the normal biomass levels produced in uncontaminated soils [41].

Studies reporting on the impact of in-field heavy metal stress on crop yield are still very limited, even though the studies reported here clearly show the impact of heavy metal pollution on crop yield, and the predominant data comes from greenhouse pot experiments or hydroponic experiments. Some experiments used irrigation water from mining sources to treat plants for biomass and yield analysis [43], and others used contaminated soils to grow plants for further analysis [44,45]. Hydroponic experiments, to test the impact of heavy metal stress on plant growth, have become an attractive method [46,47], but the method using soils (contaminated or uncontaminated) with heavy metal supplementation (spiking) remains the method of choice for crop biomass experiments. Nevertheless, most of the greenhouse experiments conducted on plants have clearly shown that heavy metal treatment (elevated levels) decreases crop yield [48,49,50,51]. Therefore, by studying the physiological and biochemical responses of plants under heavy metal stress, researchers can increase the tolerance of plants to heavy metals. In addition, by understanding the mechanisms used by plants to adapt to heavy metals, researchers could fine-tune these mechanisms to make the plants safer for human and animal consumption. These processes are regulated by signaling pathways, which consist of a reception step, a transduction step, and finally a response step.

## 4. The Role of the Cell Wall in Heavy Metal Stress Signaling in Plants

Roots serve as the main point of entry through which metal elements enter the plant system [52]. The heavy metals in the soil enter the plant root by freely diffusing through the cell wall in an unregulated manner [53]. Therefore, in the roots, this is the initial barrier for metal contact. Furthermore, the cell wall is mainly composed of cellulose, hemicellulose, and pectins [54]. The cell wall has the ability to bind heavy-metal ions in negatively charged sites (–COOH, –OH, and –SH), which results in the alteration of cell wall composition [55]. These alterations cause damage to the cell membrane, particularly to the PM. Consequently, the disruption of membrane integrity is believed to be a result of complex interactions involving functional groups of the membranes and heavy metals [56]. This is the primary site for signal perception, the downstream trigger of the defense response and the cell fate decision under heavy metal stress [57,58]. Liu et al. [59] conducted a proteomic study on *Elsholtzia splendens* cell walls under copper stress, and observed that ~40% of the differentially expressed cell wall proteins (CWPs) showed higher abundance in response to copper stress. These proteins are involved in antioxidant defense, cell wall polysaccharide remodeling, and other metabolic processes. Furthermore, the study showed that ~60% of the CWPs were in low abundance in response to copper stress, and that these proteins were involved in signaling, energy, and protein synthesis. The study identified Hsp70, small G-protein, and RAS-related GTP-binding proteins, which were all responsive to the copper stress, and which have essential roles in signal transduction. Even though the study by Lui et al. [59] clearly showed the potential role of the cell wall in downstream signaling responses under heavy metal stress, Parrotta et al. [60] stated that the intricate signaling mechanisms of the cell wall in heavy-metal responses are not well understood. Parrotta et al. [60] then highlighted the potential roles of aquaporins and wall associated kinases as two potential future targets for studying the cell wall signaling component under heavy metal stress. Indeed, Przedpelska et al. [61] observed gating of aquaporins within 10 min of heavy metal application to onion epidermal cells, irrespective of the metal applied (zinc, lead, cadmium, and mercury). The importance of aquaporins in the signaling response to heavy metals was also confirmed by Ariani et al. [62], who studied AQUA1 (a mercury-sensitive aquaporin). The results showed that a high concentration of zinc down-regulates *aqua1*. The zinc response caused the re-localization of *aqua1* into newly formed pro-vacuoles through the regulation of intracellular trafficking and post-translational modifications.

## 5. The Role of Vesicle Trafficking in Heavy Metal Stress Signaling

**Figure 1:**
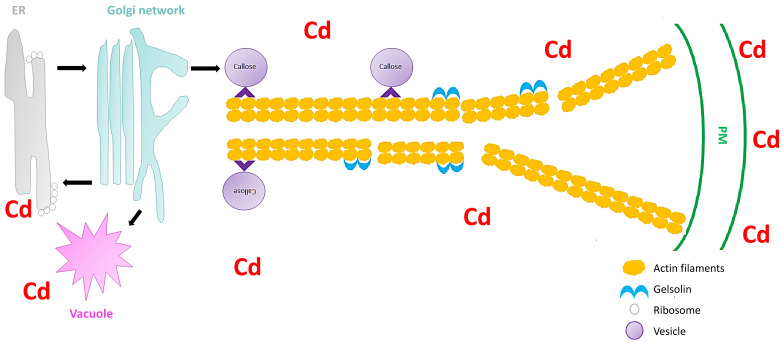
Schematic image showing the severing of the actin filaments by gelsolin under cadmium stress in *Arabidopsis thaliana* (extrapolated from Fan et al. [55]). The vesicles move (black arrow) between the endoplasmic reticulum (ER), Golgi network, vacuole, and the plasma membrane (PM). Cadmium stress triggers a callose response which are sorted and packaged in vesicles for unloading at the PM. The vesicles attaches to the molecular motors (^) which facilitate the movement on the actin filaments. However, cadmium also activates gelsolin, which cleaves the actin filaments, and therefore the vesicles cannot reach the PM and unload the callose.

Secretory vesicles are the components that transport constituents required for the construction of the cell wall. These vesicles congregate beneath the PM, and are subjected to fusion with the PM [55]. In all plant cells, intracellular transport is vital for the distribution of membrane-bound vesicles and organelles to their respective cellular destinations. Furthermore, vesicle trafficking is important for the organization of endomembranes. A disturbance in the vesicular trafficking system under heavy metal stress can lead to the disruption of cell wall construction, ultimately resulting in inhibition of cell growth [52]. Hence, it is important to understand how plant vesicular trafficking systems are influenced by heavy metal stress and how this regulation alters the cell wall signaling component. Nonetheless, vesicle-bound molecular motors are the driving force of vesicle trafficking within plant cells. These molecular motors interact with the cytoskeletal elements, microtubules, and specifically, actin filaments (localized movement of vesicles). These cytoskeletal elements are recognized as the tracks for vesicle transport. Fan et al. [55] used *Arabidopsis thaliana* to understand the significance of the cytoplasmic calcium gradient alongside actin filaments (AFs) in the vesicular trafficking system within root hairs. *A. thaliana* root hairs were subjected to cadmium treatment and fluorescence labelling with FM4-64 dye to track the impact of cadmium on endocytosis potential and membrane recycling (Figure 1). Fan et al. [55] showed that both endocytosis and vesicular trafficking were disrupted. To understand what influenced this disruption, the authors proceeded with in vivo labelling, along with laser scanning confocal microscopy, to examine the effect of cadmium on actin organization. The authors observed that the usual longitudinal arrangement of the actin filaments was altered towards a transverse arrangement, and that this alteration interrupted the vesicular trafficking system. Perfus-Barbeoch et al. [63] observed that cadmium enters the root hairs via calcium-selective channels, because cadmium has an identical charge and ionic radii to calcium. Zhang et al. [64] stated that calcium concentrations are crucial in actin filament organization, as calcium binds to gelsolin-like proteins (a calcium-dependent actin severing protein), thus activating the gelsolin, and promoting the disruption of actin filaments. Consequently, as cadmium mimics calcium, the cadmium within the cells binds to gelsolin to promote actin filament destabilization and depolymerization. Since the study revealed that the introduction of cadmium (1) disrupted the calcium gradient within the cells, (2) induced actin filament depolymerization, and (3) demonstrated a decline in vesicular trafficking, Fan et al. [55] noted that vesicle trafficking in the *A. thaliana* root hairs, were to some extent dependent on the calcium gradient and actin filament arrangement. Callose deposition at the cell wall is regarded as a defense mechanism after plants sense cadmium stress [65], and the first deposits of callose in plants were observed 5 min to several hours after contact with an inductor [66]. Therefore, because the callose synthase (CESA) is assembled primarily at the Golgi apparatus [55], the depolymerization of the AF changes the conformation of CESA and ultimately inhibits the deposition of callose in the cell wall, rendering this defense mechanism inadequate. Thus, plants require a mechanism to limit this effect of cadmium or other heavy metals on actin filaments to ensure proper callose deposition at the cell wall.

A role for pectin in the cell wall signaling cascade via the receptor-like kinase, FERONIA, was proposed by Yang et al. [67]. However, the mechanisms for pectin sensing and transducing of wall signals under heavy metals remain undiscovered. Therefore, understanding the plant vesicular trafficking events that modulate pectin at the cell wall could be a starting point to understand the involvement of pectin in cell wall signaling under heavy metal stress. Krzeslowska et al. [68] studied the mechanism of internalization of pectins in the tip of a growing apical cell protonemata of *Funaria hygrometrica* under lead stress. The authors studied the pectin epitope JIM5 (JIM5-P) because lead has a high affinity for this pectin epitope [69]. To study the internalization and vesicular trafficking of lead, Krzeslowska et al. [68] employed FM4-64 dye, and observed that vesicular trafficking intensification and common internalization of JIM5-P from the cell wall was as a consequence of lead accumulation. Furthermore, by employing the immunogold labelling method for JIM5-P identification in transmission electron microscopy, the authors observed that in the control plants JIM5-P occurred mainly within the cell wall. However, under lead stress the JIM5-P was internalized and mainly detected within PM invaginations and in different sizes of vesicles [67]. This pectin–vesicle trafficking signaling response was also observed in other plants, which could suggest that this response is a universal lead stress response [70]. The immobilization of heavy metals to cell walls via vesicle trafficking could be an important downstream plant cell tolerance reaction to stress responses. Furthermore, the internalization of these vesicles can also be internalized into endocytic invaginations of the PM, and could be transported via the endocytic or secretion pathway within plant protoplasts.

## 6. Heavy Metal Stress Signaling Events at the Plasma Membrane

After the uncontrollable movement of heavy metals through bulk flow (with water) into the root apoplastic space, the movement of the metals can go two ways: (1) The metals can move through the apoplastic space, but will be hindered by the casparian strip; or (2) the metals can move through the PM into the symplastic space. At the PM, the metals will either diffuse very slowly through the membrane, or through specific or non-specific transporters imbedded into the PM [71]. Due to the lack of transporters and receptors in the cell wall, the PM is the most likely candidate for heavy metal signal reception and downstream transduction. The PM hosts intrinsic and extrinsic membrane proteins which have diverse roles in plants. These proteins consist of a number of families of carriers and pumps responsible for metal influx and efflux. In addition, these proteins play key roles in cell adhesion, cell secretions, and cell to cell signaling events [72]. For cell only or cell to cell perception and signaling under heavy metal stress, a PM receptor is required. However, such a specific heavy metal receptor in plants remains elusive. This might be due to the different and diverse chemistry and characteristics of heavy metals, and therefore plants could not develop one-specific heavy metal receptor that can recognize all heavy metals. Moreover, ionic ligands exist, and can be transported in plants [73], but very little is known about the non-ionic ligands that can bind to metals in plants. The non-ionic ligand–metal complexes could dock into receptors, as observed in molecular modelling studies by Ananthanarayanan et al. [74], but this idea needs further investigation in plants. Nonetheless, receptors in plants have been well studied for other stresses [75,76,77].

Sensing of metals at the PM is required by the plant to mount an appropriate defense strategy. However, similarly to heavy metal receptors, which are not well explained, the sensors for heavy metals are a grey area. Nonetheless, the sensors for some of the plant nutrients are known. In *A. thaliana* the calcium sensors (CAS) are known calcium sensing receptors which are localized on the chloroplast PM [78]. The potassium transporters have been proposed as potassium PM sensors in *A. thaliana* [79]. These transporter–receptor systems are now commonly referred to as “transceptors” [80,81]. In *A. thaliana*, AtNRT1.1 is classified as a nitrate transceptor, with key roles in nitrogen sensing [82]. In addition, SULTR1;2 has been proposed to be a transceptor of sulfur in plants [83], even though the idea is still underdeveloped. Similarly, the idea of classifying IRT1 as an iron transceptor is also premature [81]. IRT1 belongs to the ZIP (Zrt/Irt-like protein) family of transporters, which are also controversially classified as potential zinc transceptors in plants [84]. Due to the fact that toxic heavy metals share similar radii with essential macro and micro nutrients, these potential transceptors could be important for sensing the heavy metals at the PM. For example, Mao et al. [85] showed that NRT1.1 may regulate the uptake of cadmium in *A. thaliana*. Furthermore, it has been well documented that cadmium uptake is regulated by IRT1, due to the similar radii of iron and cadmium [86]. A direct link between cadmium and SULTR1;2 was established by Yamaguchi et al. [87], which highlights the possible cross-talk of cadmium and sulfur. In addition, cadmium also shares similarities with zinc and calcium, therefore the possibility of sensing of cadmium by CAS and ZIPs remains to be tested.

## 7. The Role of Other Wall-Associated Proteins in Heavy Metal Signaling Events

There are proteins at the PM that are not classified as receptors, sensors, or transceptors, and which play key functional roles in heavy metal signaling. Calcium-dependent protein kinases (CDPKs) perform key roles in multiple stress responses by transducing calcium signals into downstream phosphorylation events [88,89]. In addition to a PM location, these proteins are also localized at different subcellular locations, such as the cytosol, endoplasmic reticulum, peroxisomes, the nucleus, and chloroplasts [90]. When CDPKs are activated by calcium under heavy metal stress, downstream proteins are activated, such as membrane channels, NADPH oxidase, and transcription factors, which all perform important functions individually [91]. Therefore, it is very important for plants to maintain proper CDPK levels under heavy metal stress. Xu et al. [92] conducted a proteomic study on *Raphanus sativus* seedlings under cadmium stress (10 µM and 50 µM), and observed that one *CDPK* (Unigene24993) was significantly down-regulated under 50 µM of cadmium treatment. The authors highlighted that the calcium signaling pathway might be repressed under high cadmium stress. Contrastingly, the upregulation of *CDPK* gene expression has been reported in *Cucurbita pepo* (zucchini) leaves under nickel [93], and in *Setaria italica* seedlings under chromium stress [94]. Furthermore, Yeh et al. [95] showed that CDPKs are important for mitogen-activated protein kinase (MAPK) activation under cadmium stress (up to 400 μM) and copper stress (up to 100 μM), which pointed to a link between CDPKs and MAPKs for downstream signaling under heavy metal stress.

The role of MAPKs in heavy metal stress has been well reviewed [76,96,97]. MAPKs can be found in various locations (for example, in the cytosol, the nucleus, the cell plate, microtubules, and the PM) in plant cells, depending on the isoform in question [98]. In the final step in the three tier cascade, MAPK signaling mediates the transmission of heavy metal stress signals directly to the nucleus. Guan et al. [99] overexpressed a MAPK from *Lycium chinense* in *Nicotiana tabacum* plants, and observed enhanced tolerance to cadmium stress. Furthermore, Zhao et al. [100] observed that MAPKs regulate root growth by influencing auxin signaling, and cell cycle-related gene expression in *O. sativa* exposed to cadmium stress. In another study, Xu et al. [101] used real-time quantitative polymerase chain reaction (RT-qPCR) analysis to study the responses of *MAPK* transcript to cadmium stress in *Broussonetia papyrifera* roots over time. The authors observed that the *MAPK* transcript was down-regulated at 3 h, but upregulated at 6 h, after cadmium exposure. This study highlighted the importance of MAPK recovery for downstream signaling events under cadmium stress. A longer inhibition of MAPK could be detrimental to any plant after heavy metal exposure. Nonetheless, studies have shown that membrane bound MAPK activity is often induced by reactive oxygen species (ROS), especially superoxide [76]. In turn, MAPK can also regulate ROS production in plants, often via NADPH oxidase [102]. Furthermore, after the NADPH oxidase ROS burst, MAPK could also activate downstream antioxidant enzymes under copper stress, which highlights a key role for MAPK in ROS signal amplification via antioxidant enzymes [103].

**Figure 2:**
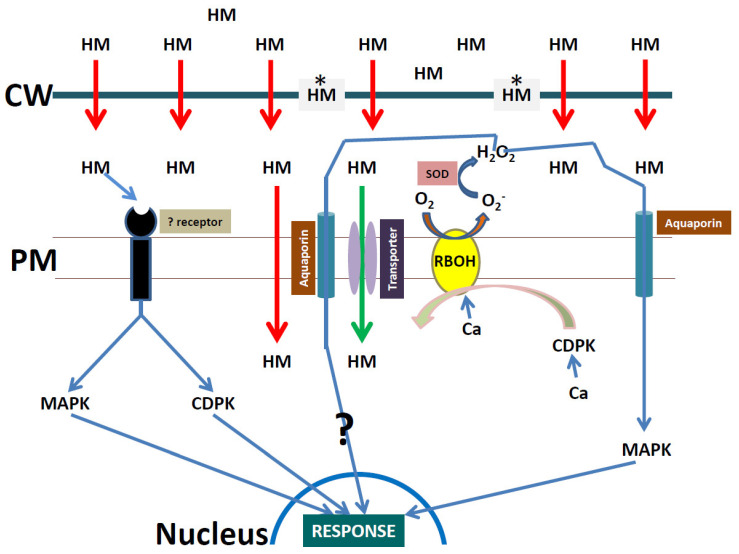
Scheme showing a putative heavy metal (HM) stress signaling cascade in plants. HMs move from outside the root through the cell wall (CW) into the apoplast by passive diffusion (red arrow). Some of the HMs are trapped (*) at the CW, but the apoplastic HMs can trigger the response of an unknown receptor, or actively move into the symplast via HM transporters (green arrow) located at the PM. HMs can also move through the PM via passive diffusion (red arrow). The unknown receptor signals responses to activate MAPK and CDPK, respectively, which can trigger a nuclear response. CDPK through calcium (Ca) activation phosphorylates NADPH oxidase (RBOH) in the presence of Ca, which induce a superoxide (O_2_^−^) burst in the apoplast. Superoxide dismutase (SOD) converts the O_2_^−^ into hydrogen peroxide (H_2_O_2_), which moves through the aquaporins into the symplast. The H_2_O_2_ can trigger a nuclear response through an unknown mechanism (?) or activate MAPK, which triggers a nuclear response under HM stress.

NADPH oxidase, which is localized on the PM, is a fundamental protein for ROS production in plants [104,105]. It utilizes NADPH as an electron donor and catalyzes the production of apoplastic superoxide radicals (O_2_^−^) after a calcium activation step [106]. The superoxide produced by NADPH oxidases regulates a wide range of biological functions and signaling cascades in plants. Plant NADPH oxidases are classified as respiratory burst oxidase homologs (RBOHs), and are homologs of mammalian phagocyte gp91^phox^ [107]. In addition to MAPK interplay, Dubiella et al. [108] showed that CDPK acts upstream of NADPH oxidases in plants. Therefore, after heavy metal sensing, CDPK could phosphorylate NADPH oxidase under optimal calcium levels, which could increase apoplastic superoxide, and which then activates MAPK for direct signaling to the nucleus (Figure 2). This hypothesis was partly supported by Chmielowska et al. [109] when the authors reviewed cadmium sensing in plants. Furthermore, Chmielowska et al. [110] showed that NADPH oxidase activity only occurred after the periods of 6 and 24 h after cadmium stress. In the same study the authors showed that a MAPK cascade gene was activated after 3 h. This could point to MAPK isoform specific activation by superoxide, or that the activation of MAPK can occur independently of superoxide, as well as a time-dependent activation of MAPK by superoxide. The NADPH oxidase cascade under cadmium stress is often described as complex when studies focus on time-courses. Indeed, the NADPH oxidase-dependent ROS burst was detected within 4 min of cadmium exposure in suspension-cultured *N. tabacum* cells [111]. Therefore, studies have pointed to two “ROS waves” under cadmium stress in plants [112,113]. Whether two “ROS waves” are activated under all heavy metal stresses remains to be tested.

## 8. The Role of Reactive Oxygen Species in Heavy Metal Stress Signaling

Reactive oxygen species (ROS) are not only harmful radicals, which are produced during stress events in plants, but these molecules also have the ability to play a signaling role at low levels [114,115]. The ROS molecules have signaling and regulatory effects on a variety of processes, such as metabolism, growth, and development in plants [116,117]. ROS have the ability to move across the PM, and can directly modulate or activate receptors or signal transducing enzymes in the cytoplasm [118]. Some ROS have a relatively short half-life, and include superoxide (2 to 4 µs), singlet oxygen (~3 μs), and hydroxyl radical (~1 μs), as well as some with a longer half-life, such as hydrogen peroxide (1 ms), which can all trigger individually or through their breakdown products [119,120]. Singlet oxygen is often referred to as an atypical ROS. The type of signaling which occurs is dependent on the type, site, and concentration of ROS present [121,122]. Furthermore, the cell surface or cell membranes of plants are often the first organelles to encounter heavy metal stress [123]. Upon the onset of cadmium stress, the pectin component of the cell wall was modified [54]. Once plants encounter heavy metal stress they require a “fast” signaling network to communicate to the rest of the organelles, in order to mount an appropriate response. These signaling networks include, but are not limited to, calcium flux, phytohormones, antioxidants, and the MAPK pathway, as well as the retrograde signaling pathway caused by ROS [124,125,126]. Furthermore, three important role players were identified as ROS signaling events, namely electric signals [127], the “calcium wave” [128,129], and the propagation of the “ROS wave” [130]. However, it should be noted that these systems do not work in isolation from one another, but actually work as part of a dynamic system.

Khare et al. [131] investigated the role of ROS signaling (response) under cadmium stress through a GeBP-LIKE 4 transcription factor (*GPL4*). The authors concluded that *GPL4*-mediates root avoidance under cadmium stress, and that this response is regulated through ROS signaling at the root tip. This study showed that ROS can directly or indirectly control transcription factors under heavy metal stress. Dang et al. [132] observed a feedback loop between a transcription factor *CaWRKY41* and hydrogen peroxide in *Capsicum annuum* under cadmium stress. The authors concluded that cadmium toxicity induces hydrogen peroxide, which in turn inhibits the activity of ROS-scavenging enzymes, leading to overaccumulation of hydrogen peroxide and ultimately upregulation of *CaWRKY41*. Many studies have analyzed the responses of genes or transcription factors on antioxidant enzyme regulation and the suppression of ROS [133,134,135].

## 9. Antioxidant Enzyme Response as a Downstream ROS Signaling Amplification Event

When trying to understand ROS signaling in heavy metal stress, the downstream modulation of antioxidant enzymes cannot be ignored. ROS signaling events upregulate the downstream antioxidant enzymes in order to ensure efficient ROS scavenging under heavy metal stress. Since superoxide is a very short lived ROS molecule in the ROS signaling event, hydrogen peroxide has often been labelled as the ROS signal amplifier in plants under heavy metal stress. For example, Liu et al. [103] performed an experiment where they exposed maize plants to copper stress and observed the results over a period of 24 h. The hydrogen peroxide concentration steadily increased from 1 h to 24 h. The same study also observed that over the same time period the activity of antioxidant enzymes (superoxide dismutase, ascorbate peroxidase, and catalase) increased gradually. The authors hypothesized that the increase in the antioxidant activity was prompted by the increase in copper concentrations and the subsequent increase in ROS concentrations. Furthermore, Ortega-Villasante et al. [136] observed hydrogen peroxide accumulation after 1 to 3 h after exposing *Medicago sativa* root cells to high cadmium and mercury concentrations. These studies support the notion that the antioxidant responses in plants are not always linear events. Factors such as species and genotypes play a major role in the antioxidant response to ROS production under heavy metal stress. 

A study by Imtiaz et al. [137] observed an increase in hydrogen peroxide content in *Cicer arietinum* with an increase in vanadium concentration. The increase in ROS content at elevated vanadium concentrations was perceived by the *C. arietinum* plants, and resulted in the response of the plants increasing their antioxidant capacity (peroxidase and catalase). Contrastingly, a study conducted in our laboratory showed that *Brassica napus* responded differently to vanadium stress [138]. This study showed an increase in superoxide content in the plants, but a decrease in superoxide dismutase activity. The increase in superoxide content was hypothesized to increase cellular damage (manifested as increased lipid peroxidation and cell death). A study by Nawaz et al. [139] observed similar findings in *Citrullus lanatus* plants exposed to high vanadium levels, where an increase in hydrogen peroxide occurred coupled with a decrease in both catalase and superoxide dismutase activities. The differences in antioxidant responses for the different plants under vanadium stress could be as a result of the sensitivity of the antioxidant enzymes to vanadium. The second hypothesis could be that the elevated ROS levels induce a negative feedback signal, resulting in down regulation of the antioxidant enzymes in certain plants species.

A study by Imtiaz et al. [140] observed the antioxidant responses of different genotypes of *C. arietinum* when exposed to high vanadium. During the experiment the authors exposed the different genotypes to two vanadium sources (ammonium metavanadate and sodium orthovanadate), as the redox state of the metal leads to different degrees of stress and response [140,141]. The study observed that the different *C. arietinum* genotypes displayed varying degrees of antioxidant enzyme (superoxide dismutase, catalase, and peroxidase) and non-enzymatic (glutathione) antioxidant activities, even though the genotypes were exposed to the same concentration of vanadium [140]. The findings showed that one of the genotypes (Noor-2009) far exceeded the antioxidant capabilities of the other genotypes, which led to increased cell viability and growth under vanadium stress. Similar observations were made in a study by Nogueirol et al. [142], which observed the effect of aluminum in two *Solanum lycopersicum* genotypes. This study observed that the genotype CNPH 0082 displayed lower concentrations of hydrogen peroxide than calabash rouge, and this was correlated with higher root and shoot catalase activity and root ascorbate peroxidase activity in CNPH 0082, which resulted in lower lipid peroxidation [142].

## 10. Amplification of the ROS Signal by Glutathione Could Improve Heavy Metal Stress Tolerance

Glutathione is a small non-enzymatic antioxidant with a half-life of ~20 min in biological fluids [143]. In plants, glutathione is synthesized by two enzymatic steps (both ATP-dependent), which involve glutamate–cysteine ligase and glutathione synthetase [144]. In the first step, the glutamate–cysteine ligase catalyzes the formation of a peptide bond between the glutamate and cysteine, and in the second step; glutathione synthase catalyzes the addition of glycine to γ-glutamylcysteine to produce γ-glutamyl-cysteinyl-glycine. Glutathione can be found in various plant organelles, such as the mitochondria, nucleus, and the cytosol. Glutathione is important in redox buffering at low concentrations, and therefore plays an important role in abiotic stresses, such as heavy metal toxicity [145]. It has also been shown to act as a disulfide reducing agent, and to assist in the protection of the thiol (–SH) group of many enzymes during signaling events under abiotic stress. Additionally, glutathione also serves as a substrate for glutathione-dependent enzymes, such as glutathione peroxidase and glutathione S-transferase, which play essential roles in the detoxification of ROS and xenobiotic compounds [146]. The concept of including glutathione as a stand-alone signaling molecule is widely debated [147], but more studies have concluded that glutathione functions downstream of ROS (i.e., hydrogen peroxide) to modulate the amplitude and duration of the upstream signals [148,149]. Indeed, Mhamdi et al. [150] and König et al. [149] both showed clear interplay between hydrogen peroxide and glutathione in the first 10 min of the hydrogen peroxide signal. Furthermore, under cadmium stress, Schützendübel et al. [151] observed that cadmium uptake was dependent on glutathione responses. The authors observed an increase in glutathione at 5 µM cadmium, and a complete depletion of glutathione at 50 µM, after 6 h. In general, glutathione has a higher affinity for heavy metals. Due to the presence of the sulfur groups in the cysteine, glutathione transports and stores reduced forms of sulfur. These reduced sulfurs bind directly to heavy metals or have an indirect action through the induction of phytochelatin synthesis [152,153]. Phytochelatins are polypeptides that are synthesized non-ribosomally from glutathione by phytochelatin synthases. These phytochelatins play important roles in plant heavy metal tolerance, as they have a high affinity for heavy metals, and can form complexes and then transport the metals to the vacuole. As a consequence, phytochelatins lowers the concentration of heavy metals in the cytoplasmic space, thus minimizing the harmful effects caused by these heavy metals [154]. It is evident that glutathione invokes multiple strategies to improve tolerance to heavy metals in plants, which include: (1) the protection of enzymes or proteins by directly scavenging ROS; (2) alleviating oxidative stress by ROS through the antioxidant enzymes glutathione S-transferase and glutathione peroxidase; and (3) synthesis of phytochelatins to minimize heavy metal uptake. Another role of glutathione was demonstrated by Talukdar [155], who concluded that an interplay exists between glutathione and hydrogen sulfide by regulation of thiol cascades during arsenate exposure in *Phaseolus vulgaris*. The glutathione and hydrogen sulfide interplay was also observed in *A. thaliana* under cadmium stress. Jia et al. [156] observed an increase in glutathione after hydrogen sulfide treatment under cadmium stress, when compared to a cadmium-only treatment. The authors also observed that hydrogen sulfide inhibited the ROS burst by inducing alternative respiration capacity and antioxidant activity. The same observations were made by Mostofa et al. [157] in *Oryza sativa* plants treated with hydrogen sulfide and cadmium. This interplay between glutathione and hydrogen sulfide points to a universal importance for the regulation of plant tolerance to heavy metal stress. This notion was proved by Kushwaha and Singh [158] who showed that in *S. lycopersicum*, *Pisum sativum,* and *Solanum melongena* seedlings that the interplay between glutathione and hydrogen sulfide regulates the mitigation of chromium toxicity. Even though studies have highlighted a link between glutathione and hydrogen sulfide in plants under heavy metal stress, these studies only focused on applying exogenous hydrogen sulfide. Therefore, the role of exogenous glutathione on endogenous hydrogen sulfide levels in plants under heavy metal stress has not been investigated.

## 11. Hydrogen Sulfide, a Multi-Signaling Molecule in Heavy Metal Stress Responses

Hydrogen sulfide is a gaseous molecule which plays an important role in many physiological processes [159]. The half-life of hydrogen sulfide in various solutions ranges from 12 to 37 h [160]. In plants, hydrogen sulfide is synthesized from various substrates in different locations. In the cytoplasm, nucleus, and mitochondria, L-cysteine is metabolized by L-cysteine desulfhydrases to produce pyruvate, ammonia (NH_3_), and hydrogen sulfide. Furthermore, if D-cysteine is present as a substrate in the mitochondrion, D-cysteine desulfhydrases will metabolise D-cysteine to hydrogen sulfide. Furthermore, in the mitochondrion, β-cyanoalanine synthase (β-CAS) catalyzes the conversion of cysteine and cyanide to β-cyanoalanine and hydrogen sulfide [161]. In the chloroplast, sulfite ions are reduced by sulfite reductase to produce hydrogen sulfide and ferredoxin. These synthesized hydrogen sulfide molecules can then be converted back to cysteine though cysteine synthase [162]. It has been suggested that hydrogen sulfide mostly signals through the persulfidation of the thiol groups of cysteine, modifying –SH to –SSH. These modifications influence protein structure, function, and activity [163]. In addition, Aroca et al. [164] showed that at least 5% of the arabidopsis proteome undergoes persulfidation. Furthermore, these persulfidated cysteines are involved in vital biological processes, such as plant growth and development, and carbon metabolism, as well as abiotic and biotic stress responses. Li et al. [165] showed that hydrogen sulfide acts downstream of hydrogen peroxide signaling in *Trifolium repens,* which points to roles in ROS amplification events. Furthermore, roles in heavy metal stress responses in plants have been elucidated for hydrogen sulfide. Liu et al. [166], showed that hydrogen sulfide alleviated zinc toxicity in *Solanum nigrum* by: (1) lowering zinc accumulation through down-regulating genes related to zinc uptake and homeostasis; (2) enhancing the expression of metallothioneins to chelate the additional zinc; and (3) altering the expression of genes related to antioxidant enzymes, to minimize oxidative stress. In a study by Ali et al. [167], exogenous hydrogen sulfide alleviated chromium toxicity in barley. Furthermore, two other studies observed similar results, where exogenous hydrogen sulfide alleviated the toxic effects of lead stress in cotton [168], nickel stress in *O. sativa* [169], and aluminum stress in wheat [170]. These studies supported the notion that hydrogen sulfide is a molecule which can confer heavy metal tolerance in plants to multiple heavy metals. Therefore, this could provide the basis for research to investigate: (1) how endogenous concentrations of hydrogen sulfide vary in crop plant species; (2) whether variations in endogenous hydrogen sulfide account for variations in heavy metal accumulation, and therefore improved metal tolerance in different plant species. Ultimately, these questions could provide insight as to which crops would be safer to plant in heavy metal polluted soils for improving both food security and food safety.

## 12. Conclusions

**Figure 3:**
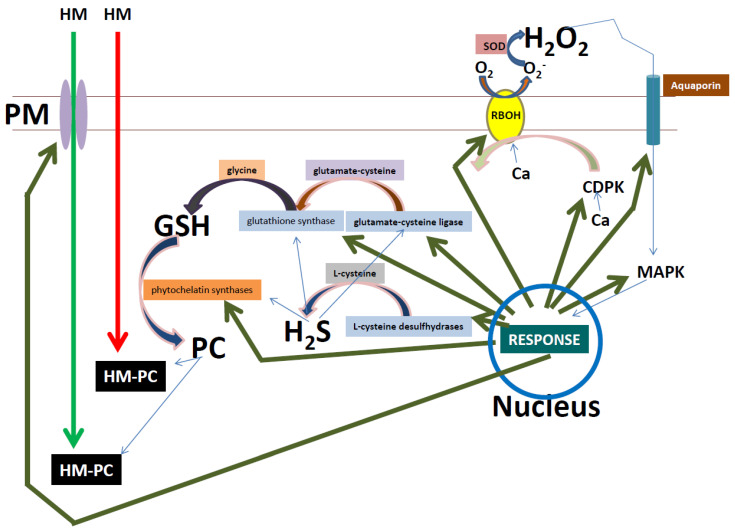
A schematic representation of the events following heavy metal (HM) reception at the PM. A nuclear activated CDPK phosphorylates and activates NADPH oxidase (RBOH) in the presence of calcium (Ca), which induces a superoxide (O_2_^−^) burst in the apoplast. Superoxide dismutase (SOD) converts the O_2_^−^ into hydrogen peroxide (H_2_O_2_), which can be transported into the symplast via aquaporins. The H_2_O_2_ can initiate a nuclear response through the activation of MAPK, which triggers downstream nuclear responses (dark green arrows) for protein synthesis. One of the products, L-cysteine desulfhydrases can produce hydrogen sulfide (H_2_S) from L-cysteine. In addition, the products glutamate–cysteine ligase and glutathione synthase can produce glutathione (GSH). H_2_S can also trigger an increase in GSH. Furthermore, another possible product, phytochelatin synthase, can convert GSH into phytochelatin (PC). The passive (red arrow) and active (green arrow) transported HM can bind to symplastic PC, forming a less toxic HM–PC complex.

Heavy metals enter plant apoplastic spaces through movement via the primary cell wall. The heavy metals can then enter the human and animal food chain by consumption of contaminated plants. These plants can be root vegetables, in which the heavy metals will concentrate after entering the root system. In addition, the heavy metals can be transported throughout the plant system from the roots into stems, leaves, flowers, and seeds, and these parts can then be consumed by humans and animals. Hence, these metals, if consumed will have severe food safety risks, and can lead to mortality. The problem of heavy metals as a food safety risk is often overlooked because of the notions that: (1) the metal concentration in the food crops are often below safety standards; and (2) that some metals must first over-accumulate over long periods of time in human and animal systems, in order to be detrimental to health. However, in this review we extrapolated from USEPA data that thallium and zirconium pose far greater risks to humans due to very low prescribed RfDo values (Table 1). We acknowledge the fact that RfDo values are not provided for lead and mercury. Nevertheless, we propose that thallium and zirconium should always be added when performing environmental and crop heavy metal assessments. In addition to food safety concerns, heavy metals also pose food security risks. Crop yields are widely affected by heavy metals in the soil, and therefore it is of utmost importance to understand plant responses to heavy metals. However, the impact of all the heavy metals on plants has not been well studied, and most studies have focused on cadmium and arsenic. Nevertheless, for root vegetables, heavy metal avoidance and heavy metal excretion (or efflux) by plants should be strategies to study. Furthermore, for leafy and seed food crops, heavy metal avoidance, heavy metal excretion, heavy metal chelation, and heavy metal sequestration should be areas for study. Plant signaling cascades are the key fundamental processes which regulate plant responses to heavy metal stress. Therefore, in this review we highlighted the key signaling events which will modulate the responses of plants to heavy metals, ultimately for the benefit of better food safety and security. From the literature it is clear that the cell wall and the apoplastic space play key roles in the initial signaling responses under heavy metal stresses. Therefore, remodeling of the cell wall is regulated by key vesicle trafficking events (from the symplastic region) that will need careful experimental investigation in the future. For example, the trafficking of pectin to the PM space can play key roles in heavy metal uptake and immobilization events. After this initial cell wall signaling event, the apoplastic side of the PM seems to be a key area for heavy metal signaling responses. We highlighted the role of plant unidentified receptors, transceptors, CDPKs, MAPKs, and NADPH oxidases in “kick-starting” the signaling cascades in response to heavy metal stress. We observed that very little is known about the signal reception step in plants under heavy metal stress, because the receptors for heavy metals are unknown. Furthermore, more data is being obtained for metal transceptors, but this field is also very young, especially with regards to heavy metals in general. We observed that more data has been released on the transduction step of the pathway where CDPKs, MAPKs, and NADPH oxidases play key roles in relaying downstream signals to trigger proper responses. We acknowledged the role of calcium in regulating all three proteins under heavy metal stress, but noted that it was widely reviewed [76,171,172,173]. Nevertheless, NADPH oxidase generates superoxide which starts the “ROS wave” under heavy metal stress. Superoxide dismutases will scavenge the superoxide and convert it into hydrogen peroxide, which we identified as a key role player in the signal transduction step. The hydrogen peroxide could enter the symplastic space potentially via aquaporins, and therefore could trigger transcription responses. In addition, the hydrogen peroxide signals could be amplified by glutathione and hydrogen sulfide, due to interaction with these two longer half-life molecules (hydrogen peroxide acts upstream of both glutathione and hydrogen sulfide) (Figure 3). This amplification cycle could include the antioxidant system, which is complex but can be regulated by hydrogen peroxide, glutathione, and hydrogen sulfide to mount the proper response, which is the last step in the signaling cascade.

## 13. Future Perspectives

**Figure 4:**
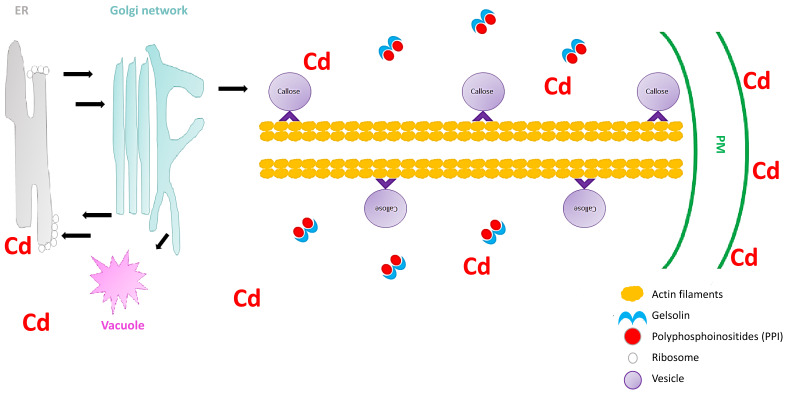
Representation of a future experiment, where gelsolin is inhibited by polyphosphoinositides (PPI) in *Arabidopsis thaliana* under cadmium stress. The vesicles move (black arrow) between the endoplasmic reticulum (ER), Golgi network, vacuole, and the PM. Cadmium stress triggers a callose response, which is sorted and packaged in vesicles for unloading at the PM. The vesicles attaches to the molecular motors (^), which facilitates the movement on the actin filaments. The induced PPI inhibits gelsolin, which subsequently cannot cut the actin filaments, and therefore the vesicles can reach the PM unhindered.

The fourth industrial revolution (4IR) started in Germany in 2011 [174] and has continued to spread rapidly to other parts of the world. Already, the increased improvement in robotics and artificial intelligence has contributed heavily to eliminating the separation of the physical world from the virtual world. Therefore, the combination of phenotyping technologies and the 4IR could increase our understanding of in-field plant responses to heavy metal stress. In order to make large scale accurate observations of heavy metal concentrations in plants grown close to contamination areas, nanotechnology sensors [175] could be used in combination with phenotyping-4IR. This would provide a large scale rapid safety assessment of heavy metal contaminated crops, and could contribute massively to providing safer foods for humans and animals. For improving plant crops to increase yield and production under heavy metal stress, a rapid understanding of the complex signaling mechanisms that govern the plant responses to the heavy metal stress is essential. Therefore, cutting-edge and high-throughput “omics” technologies, coupled with the 4IR, will be fundamental to understand the signaling processes that occur in plants under heavy metals at a definite time, and at cell-specific resolution. The high-throughput data obtained from these studies should be used in systems biology [176] to obtain the desired systems-level understanding of how plant signaling cascades govern heavy metal stress tolerance. The systems biology data could then guide bioinformatic analysis in order to increase the accuracy power of plant genetics. Therefore, CRISPR/Cas technology will be very important for dramatically accelerating the breeding of crops with improved heavy metal stress tolerance. For this purpose, the genome-wide precision and specificity of the CRISPR/Cas system should undergo massive improvements, as per the study by Tan et al. [177] on the rationally engineered SaCas9 variant (SaCas9-HF). For example, Yu et al. [178] made CRISPR/Cas mutants in *S. lycopersicum* plants by targeting the *SlMAPK3* gene, and observed improved tolerance to heat stress. *MAPK* genes are important regulators of heavy metal stress responses in plants, and therefore could be an immediate target for CRISPR/Cas studies. The genetic experiments could also include the use and transfer of minichromosomes [179], which carry the desired genes from heavy metal tolerant cultivars into heavy metal sensitive cultivars. One such gene target could be gelsolin, which increases the severing of actin filaments under heavy metal (cadmium) stress (Figure 1). Even though very little is known about the gelsolin related signaling events in plants, one future approach could be through polyphosphoinositides (PPI) induction, which inhibits actin filament severing [180]. PPI binds to the actin monomer binding site of gelsolin, and therefore gelsolin would not be able to bind to the actin filaments in plants under heavy metal stress (Figure 4). From the literature it is clear that the single-gene editing and engineering approach is still viewed as the gold standard for making plants tolerant to heavy metals. However, the dynamic environment, as well as multi-heavy metal contaminated fields, will continue to be the main problems or bottlenecks for the single-gene approach, as well as for the proposed, multi-gene approach. Therefore, the progress in the synthetic biology field towards whole-genome (de novo) assembly, editing, and transfers into plants cells (genome deplete) remains an exciting prospect for the future (see review by Liu and Stewart [181]).

## Figures and Tables

**Table 1:** Elements classified as heavy metals according to a density at room temperature of more than 5 g/cm^3^. The values for the limit in residential soils and oral reference dose were obtained from the U.S. Environmental Protection Agency (USEPA) for June 2020 [11]. The density at room temperature values was obtained from Shackelford et al. [12]. The abundance rank of the elements in the Earth’s crust was extrapolated from Anderson [13].

Elements	Limit in Residential Soils (mg/kg)	Reference Dose (RfDo) (µg/kg/day)	Density at Room Temperature (g/cm^3^)	Abundance Rank
Antimony (metallic)	31	0.40	6.70	62
Arsenic	0.77	0.30	5.73	55
Cadmium (Diet)	78	1	8.65	64
Chromium (VI)	0.31	3	7.19	21
Cobalt	23	0.30	8.90	31
Copper	3100	40	8.96	25
Iron	55,000	700	7.87	4
Lead	400	N/A	11.34	36
Manganese (Non-diet)	1900	24	7.47	12
Mercury (elemental)	11	N/A	13.53	66
Molybdenum	390	5	10.28	54
Nickel (soluble salts)	1600	20	8.91	23
Silver	390	5	10.49	65
Thallium (soluble salts)	0.78	0.01	11.85	60
Tin	47,000	600	7.31	48
Tungsten	63	0.80	19.25	53
Uranium (soluble salts)	16	0.20	19.05	50
Vanadium	390	5	6.11	19
Zinc	23,000	300	7.14	24
Zirconium	6.30	0.08	6.51	18
